# Research progress of large size SiC single crystal materials and devices

**DOI:** 10.1038/s41377-022-01037-7

**Published:** 2023-01-24

**Authors:** Xiufang Chen, Xianglong Yang, Xuejian Xie, Yan Peng, Longfei Xiao, Chen Shao, Huadong Li, Xiaobo Hu, Xiangang Xu

**Affiliations:** State Key Laboratory of Crystal Materials, Institute of novel semiconductors, Shandong University, Jinan, 250100 China

**Keywords:** Electronics, photonics and device physics, Photonic crystals

## Abstract

SiC semiconductor is the focus of recent international research. It is also an important raw material for China to achieve carbon emission peak and carbon neutrality. After nearly 20 years of research and development, we focus on the three types SiC crystals, n-type, p-type and semi-insulating, indicating the development of Shandong University for crystal growth. And defects control, electrical property, atomic polishing, and corresponding device authentication all obtain great progress. Total dislocation density of 6-inch n-type substrates decreases to 2307 cm^−2^, where BPD (Basal Plane Dislocation) lowers to 333 cm^−2^ and TSD (Threading Screw Dislocation) 19 cm^−2^. The full width at half maximum (FWHM) (0004) rocking curves is only 14.4 arcsec. The resistivity reaches more than 1E + 12 Ω·cm for semi-insulating SiC and lower than 20 mΩ·cm for n-type SiC. The impurity concentrations in 6-inch high-purity semi-insulating (HPSI) SiC crystals reach extreme low levels. The devices made of various substrate materials have good performance.

## Abstract (web summary)

The crystal growth, defects control, electrical property and corresponding device authentication of N-type, P-type and semi-insulating silicon carbide crystals in Shandong University are introduced.

## Graphical abstract

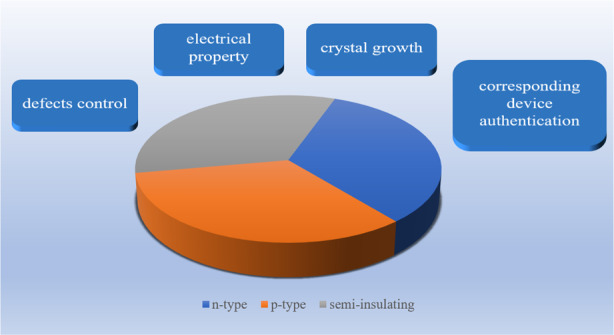

## Introduction

Silicon carbide (SiC) is a wide band gap semiconductor, and because of it has high thermal conductivity and excellent electronic properties, SiC is widely used in the manufacture of high-frequency, high-temperature, and high-power devices^1,2^. A key prerequisite for the fabrication of SiC devices is the availability of high-quality, polytypic stable and large diameter SiC substrate wafers^3,4^. Currently, the physical vapor transport (PVT) method is regarded as the most mature growth technique to obtain large SiC crystals.

The widespread use of n-type SiC is impeded by a relatively high dislocation density (about 10^3^–10^4 ^cm^−2^) in crystals used as substrates for the corresponding device structure^1^. So, the further decrease of extended defects, especially, dislocations in the crystal is crucial for improving device performance and reliability.

For semi-insulating crystals, it is very important to eliminate the foreign polytypes in the whole growth process, including nucleation and subsequent crystal growth, to further decrease defect density, otherwise, it can lead to serious quality degeneration in the field of nucleation of other defects. Also, it is imperative to remove basal plane bending and reduce the residual stresses.

Recently, p-type SiC has attracted extensive attention because of its application prospect in bipolar power and vertical electronic devices^1^. In high-voltage fields, gate turn off (GTO) thyristor and insulated gate bipolar transistor (IGBT)are mainstream devices owing to their lower dissipation^3–5^.

SiC semiconductor is the focus of recent international research. The research on SiC substrates in China has been carried out for 20 years, and realized industrialization. However, the cost of SiC substrates is still high. In addition, the low yield of SiC power devices is also an important constraint factor. Thus, the research and development of SiC materials and related devices have more work to do.

In this paper, we concentrate on the three types SiC crystals, n-type, p-type, and semi-insulating, indicating the development of Shandong University for crystal growth, defects and resistivity and corresponding device authentication. This work is supported by the State Key Laboratory of Crystal Materials and Institute of novel semiconductors. The research team actively cooperates with downstream industry or research institution to develop devices and applications, and makes contributions to the progress of SiC semiconductor in China.

### N-type SiC growth and dislocation

In the past few years, the rapid development of SiC crystal PVT growth technology has facilitated the commercial application of high-quality, micropipe-free 4-inch SiC substrates^1,2^. However, a large number of crystallographic defects, such as stacking faults and dislocations, still exist in ordinary commercial SiC single crystals^3^. Therefore, it is very important to reduce the dislocation density because it degrades the performance and long-term reliability of the device.

6-inch nitrogen doping, low resistivity 4H-SiC crystals were grown by PVT on the C-face of 4H-SiC. By optimizing the structure design and improving the temperature distribution, and based on the growth mechanism of SiC vicinal face, the low resistivity 4H-SiC single crystals with low micropipe density, stable polytype, and high structure quality were grown in the temperature field with small radial gradient. The morphology and polytype distribution of 4H-SiC crystals were investigated by Micro-Raman spectroscopy and lext-3D measuring laser microscope. It was considered that there were two types of foreign polytype transitions in the growth of 4H-SiC crystals. A polytype transition interface existed at the beginning of step, which was related to the growth mechanism of step flow. By decreasing the crystal growth rate at the beginning of step, the generation of such polymorphisms could be controlled effectively. Another polytype transition interface existed in the late growth stage^4^, which was related to the temperature rise of the growth front. This polymorphism could be effectively reduced by lowering the temperature.

**Fig. 1:**
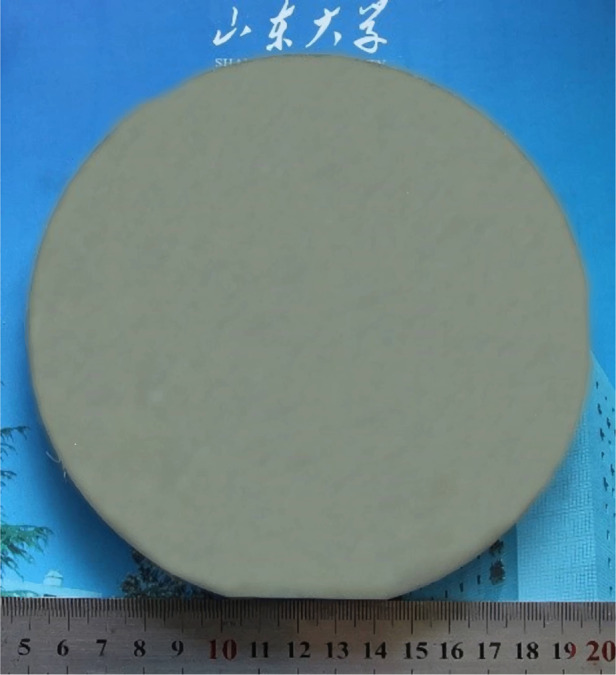
6-inch n-type SiC ingot

**Fig. 2:**
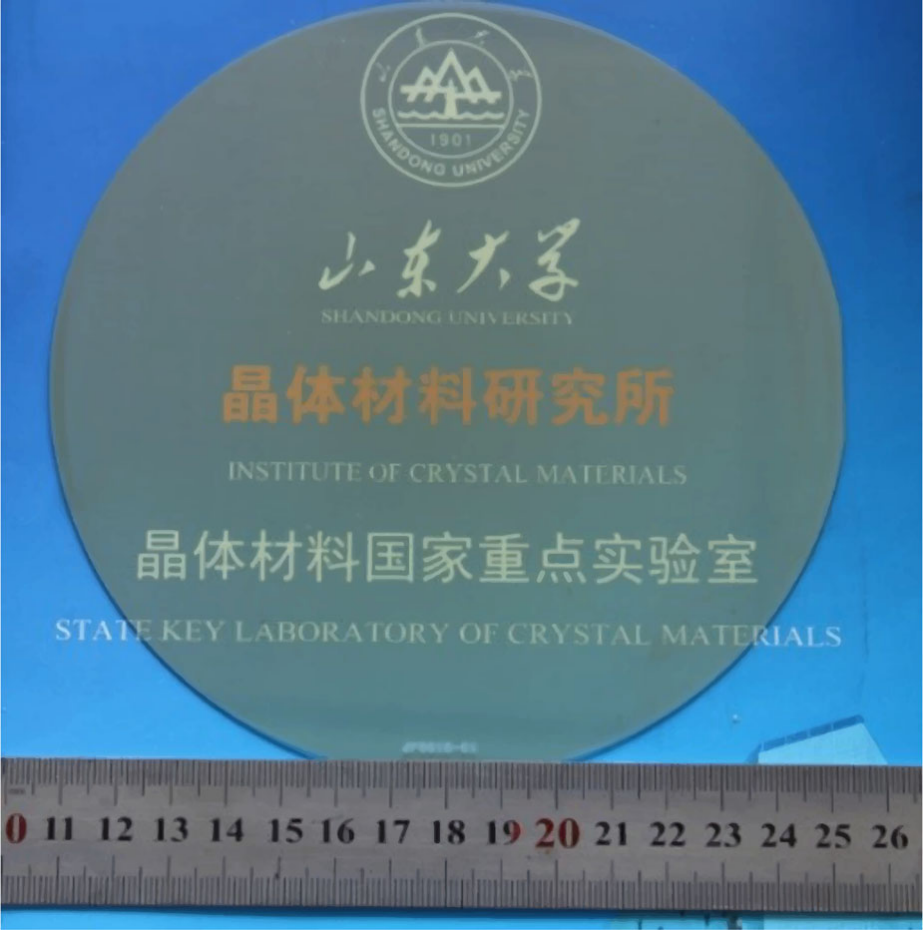
6-inch n-type SiC wafer

**Fig. 3:**
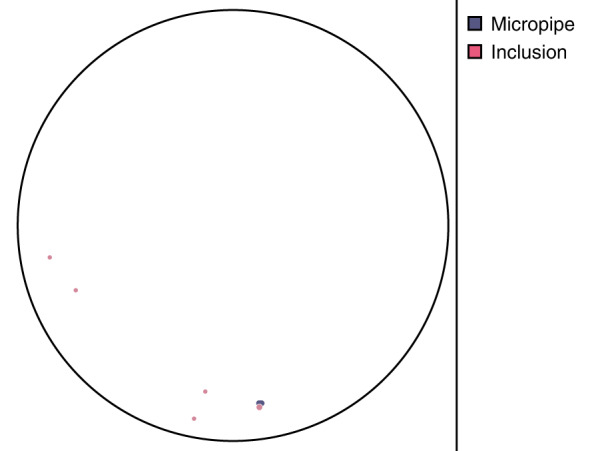
The micropipe distribution of 6-inch n-type SiC substrate (only one micropipe)

**Fig. 4:**
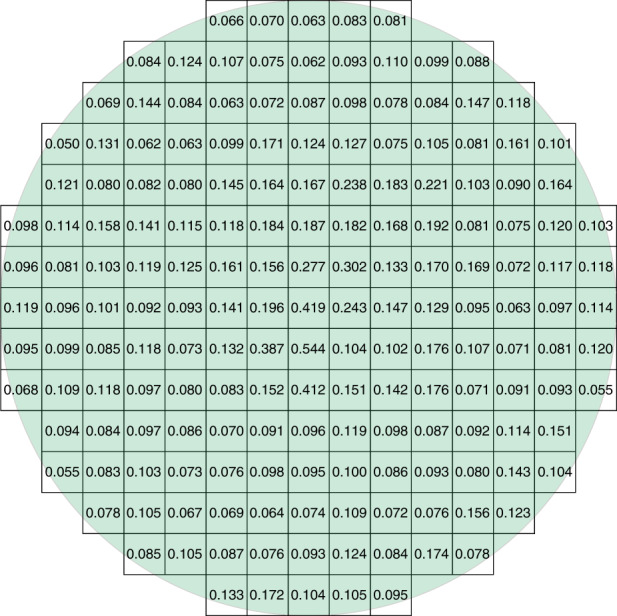
The LTV distribution of 6-inch n-type SiC substrate

Xianglong Yang et al. reported that the use of off-axis seeds in PVT growth of 4H-SiC could change the growth mechanism^5^. By observing the surface morphology of SiC single crystals grown from off-axis seeds, two growth models were proposed, one was the spiral growth induced by screw dislocation and the other was the step flow growth induced by atomic steps^5^. By adjusting the thermal field, the formation and migration of facets could be controlled. The 6-inch n-type SiC ingot is shown in Fig. 1, the substrate after CMP processing (Fig. 2), the micropipe density is lower than 0.1 cm^−2^ (Fig. 3), and 4H polytype occupies 100%. The FWHM of (0004) rocking curves is only 14.4 arcsec. The resistivity is 20 mΩ·cm with the nitrogen doping inhomogeneity <2%. The LTV distribution of 6-inch n-type SiC substrate is shown in Fig. 4, and LTV_max_ in the range of 10 mm × 10 mm is 0.546 μm.

In order to reduce the dislocation density, Xianglong Yang and Xiufang Chen et al. reported the lateral growth on patterned seeds and removed the subsurface damage of seeds, respectively^6,7^. Growth of SiC on {11–20} and {1–100} surfaces of 6H-SiC seeds by PVT method was carried out at 1700–2000 °C. The anisotropy of growth rates along different crystal directions was studied. In transverse growth, the rate in <11–20> direction was the higher, followed by <1–100> direction. The lateral growth rate could be further increased by increasing the growth temperature and decreasing the reactor pressure. In addition, compared with vertical growth, the dislocation density of channels decreased significantly during transverse growth, which proved the possibility of reduction of the dislocation density of channels^6^. 6H-SiC crystals were grown on seeds treated by different methods by PVT method. The effects of surface state and subsurface damage on the dislocation density of SiC crystals were analyzed. The dislocation density of crystals grown on seeds treated with hydrogen etching was one order of magnitude lower than that grown on seeds treated with mechanical polishing. It was considered that hydrogen etching could completely eliminate the subsurface damage of seeds^7^.

**Fig. 5:**
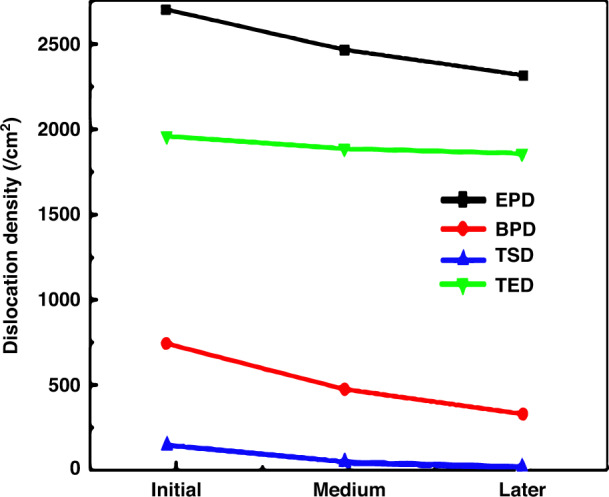
The dislocation density in the different stage of growth

**Fig. 6:**
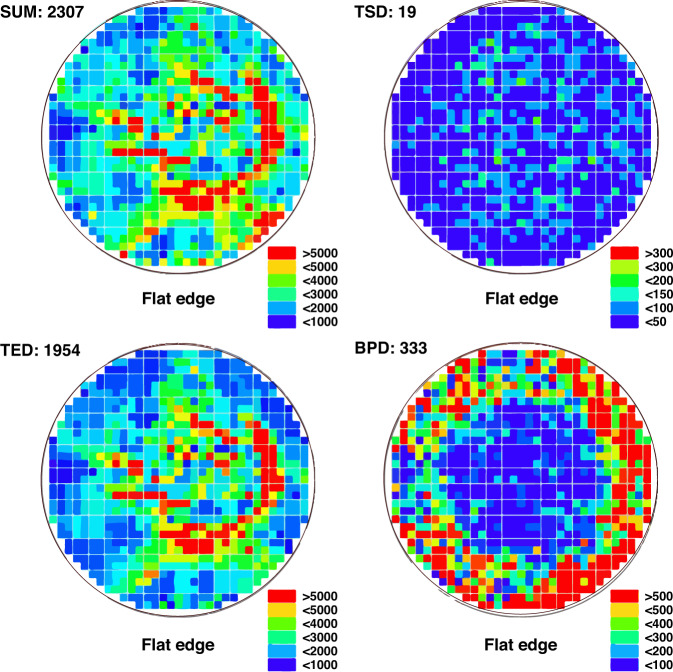
The dislocation distribution of 6-inch n-type SiC substrate

The dislocation distribution of the whole SiC wafer was obtained by scanning the SiC wafers etched by molten KOH. The dislocation distribution of SiC wafers was studied by special dislocation detector at different growth stages. The scanning etched mapping of the dislocation detector could fully display the dislocation etch pit information. According to the shape and size of the etch pits, three types of threading dislocations were identified. The dislocation detector was used to study the dislocation density and distribution of 6-inch n-type 4H-SiC crystal at different growth stages. The results show that the dislocation density decreases gradually with the growth of crystal. The total dislocation density of the wafer in the later stage of growth is reduced significantly than that in the early stage of growth, as shown in Fig. 6. TED (Threading Edge Dislocation) occupies the largest proportion in the wafer^8^. TSD and BPD exist a small proportion in the wafer, and the density gradually decreases (Fig. 5). Total dislocation density of 6-inch n-type substrates decrease to 2307 cm^−2^, where BPD lowers to 333 cm^−2^ and TSD 19 cm^−2^ (Fig. 6). It is helpful to feed back the information of the propagation and transformation characteristics of dislocation defects during the SiC crystal growth process^8^.

Cui Yingxin analyzed the effects of different nitrogen doping concentrations on lattice parameters and resistivity of 4H-SiC^9^. The structure of 4H-SiC was studied by high resolution X-ray diffractometer (HRXRD), and the lattice constants of 4H-SiC single crystals were determined. The resistivity value was tested by a non-contact resistivity testing system. It was concluded that the c - and a - lattice constants of 4H-SiC decreased, the hexagonality increased, and the resistivity decreased with the increase of nitrogen doping concentration^9^.

### P-type SiC doping characteristic

The switching speed of n-channel IGBT is faster than that of p-channel IGBT has been proved by theoretical simulation^10,11^. N-channel SiC Based IGBT devices is considered to be one of the most promising electronic devices, because it has low resistance features in the high-voltage field because of the conductance modulation. In order to manufacture high capability n-channel IGBT, p-type SiC substrates are needed as the injector region^12–14^.

2-inch p-type 4H-SiC crystals were grown by Al doping and conventional PVT method. And the N concentration in SiC was about 5E + 17 cm^−3^ as the background^15^. The interrelations of Al element dopants on resistivity and polytype were studied. The results showed that the Al atoms could be effectively incorporated into 4H-SiC crystals as well as the color of Al-doped SiC crystal was blue. With the increase of Al content in 4H-SiC single crystals, the color of Al-doped crystal became darker and eventually opaque. The mechanism of transparent crystal color development is that the crystal absorbs some wavelengths of light for atomic transition, and the remaining unabsorbed light is transmitted to show the color of the crystal. The band gap width of intrinsic 4H-SiC is 3.26 eV. Visible light >380 nm can be transmitted, and ultraviolet light <380 nm can be absorbed. A small amount of Al impurities will introduce an acceptor level of 0.19 eV into 4H-SiC, which will fully absorb the wavelength above 1500 nm and below 380 nm, and partially absorbs the light in the range of 380–1500 nm. However, with the increase of doping concentration, the discrete Al impurity energy levels are split into energy bands, the range of light being fully absorbed moves to low wavelengths (that is, the wavelength of light being fully absorbed changes from more than 1500 nm to more than 600 nm, or even smaller), and the rest of the partially absorbed light (mainly blue light with a wavelength rang of 435–480 nm) is also absorbed enhanced. Finally, the transmittance of light decreases, and the crystal appears opaque. Under the condition of heavy Al doping, the polytype of 4H-SiC was unstable and 6H-SiC polytype appeared. N is the donor for doping SiC and substitutes the C lattice sites of SiC. B is the acceptor for SiC doping and occupies Si/C lattice sites. Al is the acceptor for SiC doping and occupies Si lattice sites. The atomic radii of N and B are both smaller than that of Si, and the incorporation into the crystal will lead to lattice contraction. The atomic radius of Al is larger than that of Si, and the incorporation into the crystal will lead to lattice expansion. When N or B is doped into SiC crystal, it has the effect of neutralizing the lattice expansion caused by Al atoms and stabilizing the atomic arrangement order of the crystal. Therefore, on the premise of ensuring the crystal quality, the doping concentration of Al element can be increased and the crystal resistivity can be reduced. In order to eliminate the polytype of 6H, Al-N co-doping was carried out. And results showed the polytype of p-type 4H-SiC single crystals returned to be stable^15^. However, owing to the difficulty in effectively to command the release of Al, Al-N co-doped 4H-SiC turned to n-p-n type. It was difficult to keep the high quality and low resistivity at the same time. Noncontact resistivity measurement showed p-type 4H-SiC wafers has the minimum resistivity of about 4 Ω·cm^15^, this resistivity value was a little high.

In order to continue to reduce the resistivity, p-type 4H-SiC crystals were grown by using Al-B co-doping technique, reported by Xuejian Xie et al.^16^. By adjusting the doping ratio of Al and B, p-type 4H-SiC single crystal without 6H polytypes were obtained. Similar to Al-N co-doping, the Al-B co-doping method also introduced small lattice distortion to 4H-SiC, which promoted the stabilization of the 4H-SiC polytype. Because the B element was the acceptor atom, and different from N element with donor atom, thus the lower resistivity was realized. The lowest resistivity of p-type 4H-SiC achieved 0.495 Ω·cm, in this case the doping concentration of Al and B were 2E + 19 cm^−3^, 4.7E + 17 cm^−3^, respectively^16^. To achieve lower resistivity, the Al and B concentrations should continue to be optimized, and the background N impurities should be minimized. The Al-B co-doping technique paved a new way for growing high-quality and low-resistivity p-type 4H-SiC, and played an important role in the development of high-voltage power electronic devices^16^. However, because of the memory effect of B element, it was still to develop and optimize the release of Al to obtain the high uniformity and low resistivity. According to the above proposal, 4-inch p-type SiC single crystals were grown with Al-B co-doping technique. Raman spectroscopy mapping test showed that p-type SiC was entirely 4H-SiC and no others polytype. The FWHM of (0004) X-ray rocking curve was <30 arcsec, implying the high crystalline quality of p-type 4H-SiC. Resistivity mapping results showed that the resistivity deviation of the obtained p-type SiC substrates was 23.51%, and the lowest resistivity achieved 0.30 Ω·cm^16^.

Because devices manufactured on p-type SiC substrates are usually power electronic devices with high current densities, it is significant to study the lattice vibration properties to help enhance the reliability of devices^17^. The Raman spectra of p-type 6H-SiC with different Al doping concentrations were investigated in the temperature range of 203–653 K^18^. Results exhibited all Raman peaks showed redshift and broadened with temperature rising. The E2(low) as well as E2(high) modes were smaller correlation on the Al doping contents, while A1(LO) mode exhibited a strong correlation of Al doping contents^18^. And the different characteristics of A1(LO) mode from E2(low) and E2(high) modes in heavily Al doped 6H-SiC showed that the A1(LO) mode was dominated by the thermal expansion and anharmonic effect as well as the acceptor ionization effect. Compared to other phonon modes of Raman spectra, the A1(LO) lifetime was more sensitive to the Al concentration and the intensity decreased with the increasing temperature^18^.

### Semi-insulating SiC growth, stresses, and electrical properties

Due to the lack of large-size and high-quality homogeneous GaN substrates, sapphire, SiC, Si, and other foreign substrates are widely used in GaN-based HEMTs (High Electron Mobility Transistor)^19,20^. Among them, semi-insulated SiC is still the best choice for substrate due to its high thermal conductivity, high resistivity, and maturity in mass production of 6-inch large wafers^21,22^. For semi-insulating SiC, the polytype, basal plane bending, residual stress, and resistivity are main problems that need to be solved.

Yang Xianglong et al. used PVT method to grow 4H-SiC on on-axis seeds and analyzed the formation and transformation of polytype during the growth process^23^. It was found that at the initial stage of growth, heterogeneous nucleation occurred easily due to the high supersaturation in the periphery of the crystal plane, because the transverse growth of the 4H region could not extend to the periphery of the crystal plane. As the growth progressed, 4H-SiC in the central region became dominant and expanded to the edge, and the extraneous polytypes overlapped with 4H-SiC in the central region and gradually covered it^23^.

The effect of seed mounting methods on the bending degree of the basal plane in 4H-SiC grown by PVT method was analyzed by HRXRD^24^. The bending degree of the basal plane of the crystals grown by seeds in the open state was obviously better than the closed state. It was suggested that high-quality crystals could be grown from low-quality seeds by optimizing growth conditions^24^. Combined with the corrosion results of molten KOH, the effects of macroscopic shear stress and structural defects caused by the difference of thermal expansion coefficient between SiC seeds and graphite holder on the bending of 4H-SiC single crystal basal plane were discussed. The influence mechanism of dislocations was also discussed^24^.

The residual stresses in 4H-SiC bulk crystals were investigated by neutron diffraction method in three orthogonal directions (<0001>, <11–20> and <1–100>), and the stress and strain were quantitatively calculated by using Bragg equation and Hooke’s law^25^. The strain ranged from 10^−4^ to 10^−3^ along these three directions, while the stress was anisotropic. The stress along <0001> direction was compressive stress at the secondary flat side, and tensile stress on the opposite side. Along the direction <11–20>, the stress was compressive and relatively uniform, ranging from −763 to −490 MPa. However, along the direction <1–100>, the stress was tensile stress, and the stress gradient from the primary flat side to opposite side was as high as 17 MPa mm^−1^, and the stress range was 673–2953 MPa. Therefore, it was considered that the crystal was prone to cracking in this direction^25^. It was proposed that the crystal with lower growth rate had less stress. Moreover, once stress was generated at the nucleation stage, it was inherited during subsequent growth^25^.

**Fig. 7:**
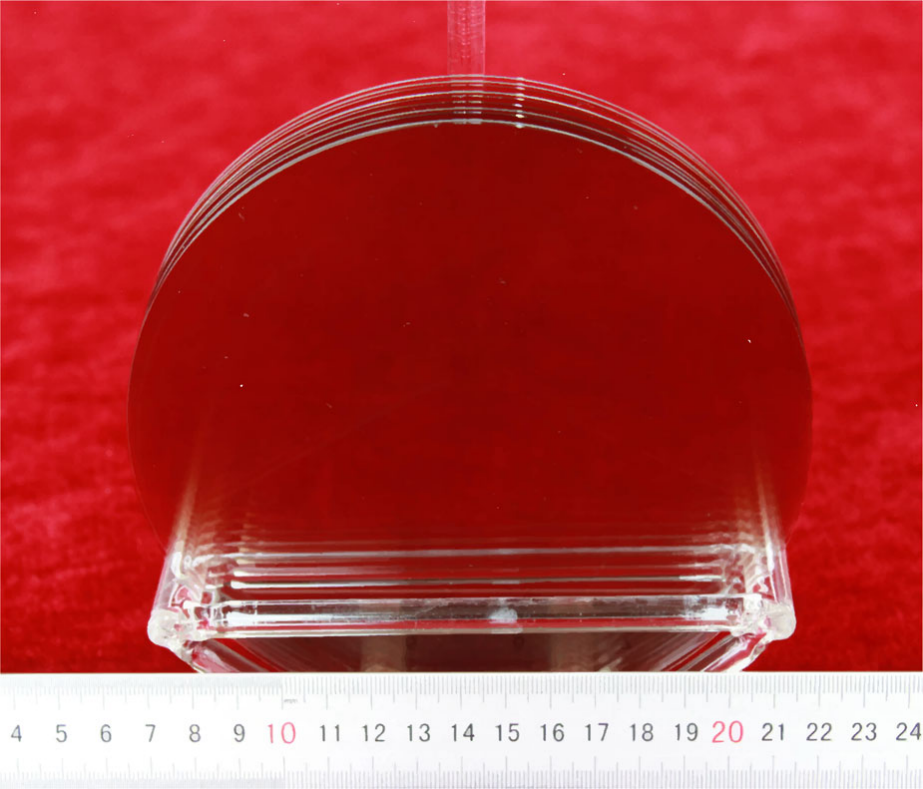
6-inch HPSI SiC substrates

**Fig. 8:**
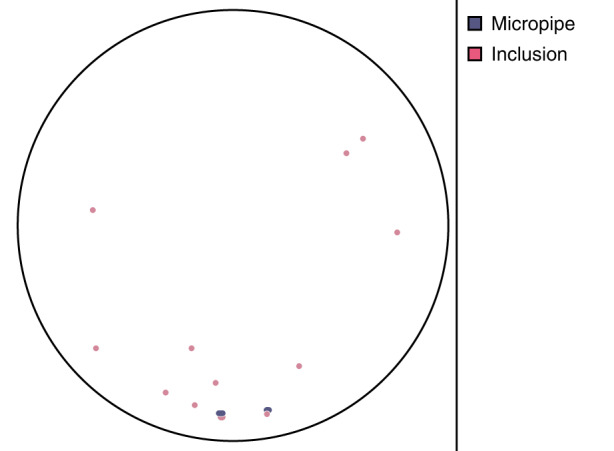
The micropipe distribution of 6-inch HPSI SiC substrate (only two micropipes)

**Fig. 9:**
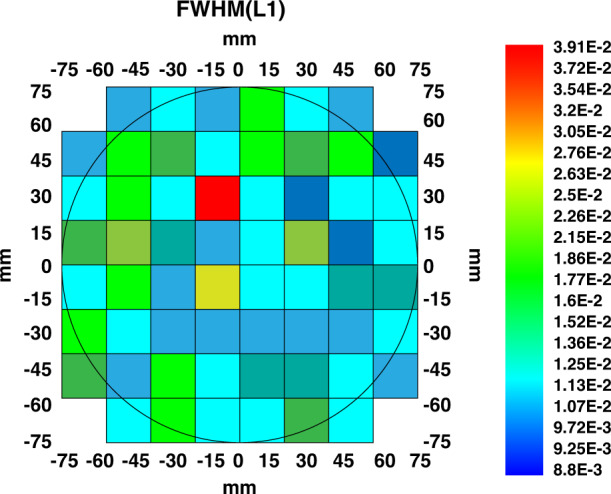
The FWHM distribution of 6-inch HPSI SiC substrate

**Fig. 10:**
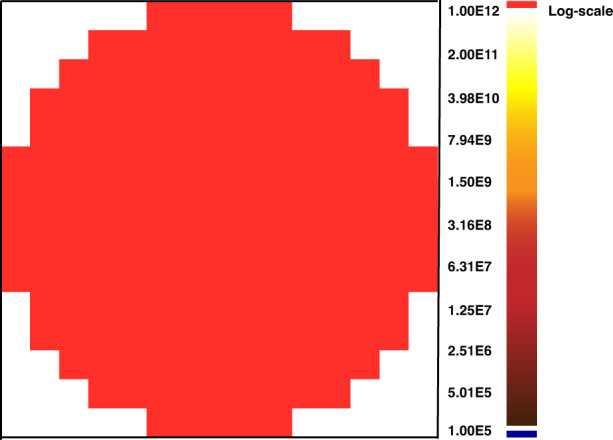
The resistivity distribution of 6-inch HPSI SiC substrate

6-inch HPSI SiC crystals were obtained by above optimizing temperature field eliminating 6H or 15R polytype, improving seed crystal fixation and reducing residual stress. The HPSI SiC substrates are shown in Fig. 7. The micropipe density by optical microscope is reduced lower than 0.5 cm^−2^ (Fig. 8), and 4H crystal type ratio is 100%. The results of (0004) rocking curves indicate high crystalline quality and virtually flat basal planes, the FWHM of the whole wafer is uniform, except one dot is slightly higher, as shown in Fig. 9. The resistivity reaches more than 1E + 12 Ω·cm in the whole wafer area (Fig. 10). The impurity concentrations in 6-inch HPSI SiC crystals are as follows at the stage of growth process (Table 1).

**Table 1:** The impurity concentration measured by SIMS in 6-inch HPSI SiC crystals at different stage of growth process

Element	Concentration at the initial stage/cm^3^	Concentration at the medium stage/cm^3^	Concentration at the later stage/cm^3^
N	8.1E + 15	5.2E + 15	3.4E + 15
B	4.2E + 15	3.7E + 15	3.2E + 15
Al	<1E + 15	<1E + 15	<1E + 15

### Epitaxial growth and device results

AlGaN/GaN HEMTs have many excellent properties, such as large band gap energy, high electron saturation rate, high critical electric field strength, and the sheet charge density at the interface is high due to the large offset and strong polarization effect of the conductive band. Therefore, AlGaN/GaN HEMTs have great application prospects in high-power microwave devices and high-frequency devices^26,27^. AlGaN/GaN with high electron mobility was grown by metal-organic chemical vapor deposition (MOCVD) method on 4-inch semi-insulating 4H-SiC substrates^28^. X-band microwave power high electron mobility transistors were made. The hall mobility was 2291.1 cm^2^/(V·s), and the two-dimensional electron gas density was 9.954E + 12 cm^−2^ at 300 K. The maximum drain current density was 1039.6 mA/mm and the peak external transconductance was 229.7 mS/mm for the HEMT device with a gate length of 0.45 μm. The f_T_ and f_max_ values measured on the device were 30.89 GHz and 38.71 GHz. Uncooled devices showed high linear power gain of 17.04 dB and high power-added-efficiency of 50.56% at 8 GHz with drain bias (−3.5,28) V. In addition, when the drain bias was (−3.5,40) V, the saturation output power density was 6.21 W/mm, and the power-added efficiency was 39.56% and the power gain reached to 11.91 dB^28^.

In 2018, Shandong University made photoconductive switch by the same electrode with the HPSI 4H-SiC. The conduction resistance was <1 Ω, when the triggering wavelength was 355 nm, the triggering energy was 10 mJ, and the bias voltage was 6 kV^29^. In the same year, China Academy of Engineering Physics in cooperation with Shandong University, prepared a 4H-SiC photoconductive switch and discovered a microwave oscillation phenomenon with an oscillation frequency of 1 GHz^30^. This was the first time that the microwave oscillation caused by the characteristics of the material itself was found based on the SiC photoconductive switch.

Semi-insulating SiC could be used for other devices, such as UV optoelectronic devices^31^, GaN-based long wavelength light-emitting diodes^32^. Graphene was grown on semi-insulating 4H-SiC (0001) by thermal decomposition^33–36^. Graphene could effectively reduce the biaxial stress of GaN films, and the strain relaxation could improve the incorporation of indium atoms in InGaN/GaN multi-quantum wells (MQWS), resulting in a significant red-shift in the emission wavelength of InGaN/GaN MQWS^32^.

The I-V characteristics of typical Schottky Barrier Diodes (SBDs) lie in its lower forward voltage drop and reverse leakage current, as well as its better temperature performance. Standard 600 V SiC SBD (Schottky Barrier Diode) epitaxial process was performed on Epi-Ready N-type 4H-SiC substrates^37^. The specific structure was 0.5 μm thick N-type buffer layer with a doping concentration of 1E + 18 cm^−3^, and 6.0 μm thick N-type drift layer with a doping concentration of 1E + 16 cm^−3^. The surface roughness RMS was only 0.258 nm. A 600 V/50 A SiC Schottky diode with a single chip area of 4.2 mm × 4.2 mm was developed. When the conduction current was 50 A, the forward voltage was 1.9 V, and the reverse voltage was 600 V, the leakage current was <50 μA. The DC performance of the device closed to that of the imported substrate^37^.

## Conclusion

After nearly 20 years of research and development, 6-inch high quality n-type and semi-insulating SiC crystals and 4-inch p-type SiC crystals were grown. Total dislocation density of 6-inch n-type substrates decreased to 2307 cm^−2^, where BPD lowered to 333 cm^−2^, the FWHM of (0004) rocking curves was only 14.4 arcsec. The micropipe density of 6-inch n-type and semi-insulating SiC crystals decreased to <0.5 cm^−2^. The resistivity reached more than 1E + 12 Ω·cm for semi-insulating SiC and lower than 20 mΩ·cm for n-type SiC. The impurity concentrations in 6-inch HPSI SiC crystals were reduced to extreme levels. The HEMT devices made from semi-insulating SiC substrates exhibited a saturation output power density up to 6.21 W/mm at 8 GHz, with a power gain of 11.91 dB and a power-added efficiency of 39.56%. The photoconductive switch made from HPSI 4H-SiC demonstrated good performance indicators. Standard 600 V SiC SBD fabricated with n-type SiC substrates exhibited good performance. Semi-insulating SiC could be used for UV optoelectronic devices and GaN-based long wavelength light-emitting diodes.
